# Nutrition and Regulation of Muscle Protein Synthesis

**DOI:** 10.3390/nu15184017

**Published:** 2023-09-16

**Authors:** Sara Salucci

**Affiliations:** Cellular Signalling Laboratory, Department of Biomedical and NeuroMotor Sciences (DIBINEM), University of Bologna, 40126 Bologna, Italy; sara.salucci@unibo.it

Skeletal muscles are an indispensable actor for daily activities, playing an essential role in locomotion through both the control of posture and position and by joint stabilization. In addition, they are involved in body temperature maintenance and constitute nutrient reserves. Skeletal muscle represents approximately 40% of human body weight, but this percentage physiologically decreases when tissue undergoes atrophy. During aging, a decline in muscle strength and mass can be observed and muscle mass decreases by approximately 3–8% per decade after 30 years of age and this reduction appears significant after 60 years [1]. Muscle mass loss with age is associated with a variety of clinical features which include alterations in body glucose homeostasis, falls, fractures, disability, and chronic diseases. Moreover, skeletal muscle dysfunctions or genetic abnormalities, such as myopathies, dysphagia, ataxia, weakness, tremors, and tendon ruptures, can lead to muscle mass reduction. Skeletal muscle mass depends on a fine balance between protein synthesis and protein degradation, and several signals, including oxidative, mechanical, and nutritional stimuli, as well as cytokines or growth factor, are involved in the modulation of the signaling pathways that control protein and organelle turnover [2]. Cancer, infections, diabetes, organ failure, or disuse promote the loss of proteins, organelles, and cytoplasm, leading to muscle atrophy. Therefore, the maintenance of the correct biogenesis and biosynthesis appears necessary in defining the size and function of muscle cells [2]. In this scenario, nutrition intake represents a potential clinical intervention since it can reactivate protein synthesis during atrophy. In this regard, some epidemiological studies have indicated strategic diets with a sufficient intake of beneficial foods for preventing sarcopenia, a condition characterized by the loss of skeletal muscle mass and function with advancing age [3]. Sarcopenia comprises some common muscle features including a decrease in muscle mass, a reduction in the size and in the number of myofibers, as well as in in the number and function of satellite cells. The latter, known as resident muscle stem cells, play a crucial role in muscle regeneration and in maintaining muscle mass during aging. Satellite cells are localized at the periphery of the muscle fiber, between the basal lamina and sarcolemma; they are quiescent under normal conditions, and they are activated after muscle damage [4]. Several studies associated a low level of vitamin B6 with sarcopenia and, in particular, an original paper published in this Special Issue demonstrated that vitamin B6 deficiency induces satellite cell dysfunctions [5]. Vitamin B6 is involved in amino acid metabolism, and it can be considered a potential antioxidant for cells. Komura et al. (2023) [5] showed that a low vitamin B6 uptake led to an impaired satellite cell synthesis or reduced satellite cell proliferation capacity in skeletal muscle mice, thus contributing to sarcopenic phenotype development.

With regard to sarcopenia, Shin et al. (2023) [6] discussed the role of Panax ginseng berry extract (GBE) in sarcopenic obese mice. Sarcopenic obesity, the combined state of sarcopenia and obesity, leads to higher risks of metabolic diseases and disability and therapeutic strategies to tackle this disease are still lacking. The authors demonstrated that GBE, obtained from the root of Panax ginseng, a known medical herb with anticancer, antidiabetic, anti-obesity, and anti-inflammatory properties, could improve this pathologic condition. In fact, GBE acted by restoring the correct balance between protein synthesis and degradation through promoting the activation of the PI3K/Akt pathway in skeletal muscle mice. As is known, the upregulation of PI3K and Akt leads to a significant increase in the phosphorylation of all mammalian targets correlated to protein synthesis. Therefore, GBE administration in sarcopenic obese mice significantly increased muscle mass, increased the myofiber cross-sectional area, and decreased adipose tissue weights and adipocyte size, as well as increasing the production of inflammatory cytokines in the muscle tissue [6].

In their paper, Jeun and Choung (2023) [7] described the potential role of oyster hydrolysates in preventing muscle atrophy. Oyster hydrolysates, marine food resources containing high-quality proteins, show antioxidant, anti-inflammation, and anti-cancer activities. Thanks to these properties, Jeun and Choung demonstrated the ability of these compounds to prevent the C2C12 myotube diameter reduction induced by dexamethasone treatment. Oyster hydrolysates stimulated protein turnover, particularly PI3K/Akt pathway activation, and mitochondrial biogenesis [7].

Another high-quality protein that contributes to the maintenance of skeletal muscle mass is egg white protein (EGG), an arginine-rich protein source with a lower amount of leucine. Koshinaka et al. (2023) demonstrated that EGG supplementation in rats had a higher efficiency in muscle gain compared to other common animal-based proteins [8].

Continuing the discussion of dietary protein as an important strategy to manage and prevent sarcopenia, Lees et al. (2023) [9], in their original paper, considered fish protein hydrolysates (FPH) as high-quality sources of dietary protein. The authors demonstrated the ability of FPH to promote skeletal muscle synthesis activation in the skeletal muscle cell model, suggesting that FSH could be considered an alternative source of readily bioavailable protein to support skeletal muscle health and anabolism in older people [9].

This Special Issue also includes a paper evaluating rosemary leaf extract activity in primary skeletal muscle cells [10]. Rosemary leaf extract is a source of phenolic compounds and shows interesting biological properties, including antidiabetic, anti-inflammatory, antioxidant, and anticancer benefits. Its antioxidant activities are mainly attributed to a phenolic diterpene known as carnosol. Morel and coworkers (2023) [10] demonstrated carnosol’s ability to activate the signaling pathways involved in the control of skeletal muscle hypertrophy and atrophy. Carnosol inhibited both proteosome and protein degradation activity and, in this way, it appears useful for the treatment or prevention of skeletal muscle atrophy.

Among the original articles published in this Special Issue, Salucci et al. (2023) [11] described the effect of curcumin on rhabdomyosarcoma, a malignant tumor of striated muscle. It originates from mesenchymal precursors that, under normal conditions, differentiate into skeletal muscle cells, but in presence of aberrations they fail to complete myogenesis [12,13]. Curcumin, thanks to its anticancer properties, leads to cell cycle arrest, inhibits cell migration, and induces apoptotic cell death involving the AKT-mammalian targets of rapamycin (mTOR), AMP-dependent kinase (AMPK), and p53 [11].

Finally, this Special Issue contains two reviews that recognize nutritional interventions as an important strategy to manage and prevent skeletal muscle loss. In this regard, Massini and colleagues (2023) [14] discussed the nutritional and dietary strategies useful in preventing or delaying the loss of muscle mass, a complication that can frequently be observed in patients with chronic kidney disease (CKD). In the review, the authors suggested that low-protein diets supplemented with essential amino acids and ketoacids could have beneficial effects in preserving muscle mass and slowing the progression of CKD [13]. Furthermore, Salucci and coworkers (2023) [15] underlined the role of extra virgin olive oil (EVOO) in maintaining skeletal muscle homeostasis during aging. The data collected suggest that EVOO, thanks to its phenolic content, can activate anabolic pathways and counteract mitochondrial alterations and inflammatory processes. 

In conclusion, this Special Issue in *Nutrients*, titled “Nutrition and Regulation of Muscle Protein Synthesis”, highlights the strategic role of nutritional approaches, based on supplementation with vitamin (B6), herbal extract (GBE), phenolic compounds (carnosol, EVOO, curcumin), and high-quality dietary proteins (oyster hydrolysates, EGG, FPH), in the prevention of muscle mass loss associated with diseases or aging.

The Guest Editor warmly thanks all of the authors for their contributions to this Special Issue.
